# Silver Fir (*Abies alba* L.) Polyphenolic Extract Shows Beneficial Influence on Chondrogenesis In Vitro under Normal and Inflammatory Conditions

**DOI:** 10.3390/molecules25204616

**Published:** 2020-10-11

**Authors:** Mateja Sirše, Samo K. Fokter, Borut Štrukelj, Janja Zupan

**Affiliations:** 1 Department of Orthopaedics, University Medical Centre Maribor, Ljubljanska ulica 5, 2000 Maribor, Slovenia; samo.fokter@guest.arnes.si; 2 Department of Pharmaceutical Biology, Faculty of Pharmacy, University of Ljubljana, Askerceva 7, 1000 Ljubljana, Slovenia; borut.strukelj@ffa.uni-lj.si; 3 Department of Clinical Biochemistry, Faculty of Pharmacy, University of Ljubljana, Askerceva 7, 1000 Ljubljana, Slovenia; janja.zupan@ffa.uni-lj.si

**Keywords:** mesenchymal stem/stromal cells, polyphenols, chondrogenesis, inflammation, subchondral bone, osteoarthritis

## Abstract

Several plant polyphenols have been shown to reduce osteoarthritis symptoms due to their antioxidant, anti-inflammatory and immunomodulatory properties. We investigated the effects of two different polyphenolic extracts (Belinal, Pycnogenol) and two different polyphenols (resveratrol, quercetin) on the chondrogenic potential of bone-derived mesenchymal stem/stromal cells (MSCs) from healthy donors and patients with osteoarthritis. Our main aim was to determine whether Belinal, a commercially available polyphenolic extract from silver fir (*Abies alba* L.) branches, has comparable chondrogenic potential with the other tested extract and the polyphenols under inflammatory and non-inflammatory conditions. In our study, Belinal promoted significantly greater chondrogenesis compared to the untreated (*p* = 0.0289) and resveratrol-treated (*p* = 0.0468) MSCs from patients with hip osteoarthritis under non-inflammatory conditions. Under inflammatory conditions, chondrogenesis was significantly enhanced for MSCs treated with Belinal compared to the control (*p* = 0.0483). The other extract and the polyphenols did not show any significant effects on chondrogenesis under non-inflammatory or inflammatory conditions. None of the tested extracts and polyphenols showed significant effects on chondrogenesis in healthy donors, under either non-inflammatory or inflammatory conditions. Our data show that Belinal can boost the chondrogenesis of MSCs derived from patients with osteoarthritis, under both non-inflammatory and inflammatory conditions.

## 1. Introduction

Osteoarthritis is the most widespread, chronic, progressive and debilitating joint disease of the elderly population, and as such, it represents a huge socio-economic burden [1]. Therefore, many efforts have been made to alleviate the symptoms and slow the progression of this disease. Osteoarthritis is characterised by the progressive destruction of the articular cartilage, accompanied by synovial inflammation and changes in subchondral bone and peri-articular muscle and ligament tissue [2].

A growing body of research shows that the complex aetiology of osteoarthritis comprises genetic, mechanical and inflammatory factors [2]. Osteoarthritis results in the irreversible loss of articular cartilage, which has little intrinsic capacity for repair due to its avascular nature and low mitotic activity [3]. This cartilage loss leads to pain, physical disability, movement restriction and morbidity.

Pro-inflammatory cytokines are critical mediators of cartilage breakdown; however, it is not yet completely known whether it is the damage to the cartilage that triggers inflammatory responses, or vice versa [4]. Various treatment strategies for cartilage regeneration have been proposed, although to date these have been without optimal outcomes. Currently, the most widespread treatment for osteoarthritis is symptomatic, such as pain management and total joint arthroplasty. Regenerative medicine, on the other hand, brings new hope for cartilage repair through the activation of the endogenous stem/progenitor cells [5].

Mesenchymal stem/stromal cells (MSCs) are multipotent progenitors that reside in the various musculoskeletal tissues, also in the adult organism [6]. MSCs can regenerate cartilage, bone, muscle and other connective tissues following trauma or injury [5,7,8]. Recent studies have revealed that bioactive compounds that naturally occur in seaweed, herbs, fruit and vegetables can modulate the self-renewal and differentiation potential of adult stem cells [9,10]. However, there is evidence that endogenous MSCs can become physiologically underproductive as a result of aging [11] or degenerative disorders, such as osteoarthritis [12]. On this basis, new strategies to boost the regenerative capacity of the endogenous MSCs are of primary focus in regenerative medicine research.

As inflammation has been shown to have a pivotal role in cartilage degradation, particular interest has focused on the anti-inflammatory actions of natural compounds, such as polyphenols. Polyphenols represent a wide variety of compounds that are found in fruit, vegetables, red wine, tea, plant oils, cocoa and other sources. Their antioxidant, anti-inflammatory and immunomodulatory properties have been studied under many chronic inflammatory conditions, including osteoarthritis [13,14]. These studies have shown that polyphenols can inhibit the expression and release of a number of pro-inflammatory mediators and proteolytic enzymes in chondrocytes in vitro. In vivo studies of animal models of osteoarthritis have shown reduced tissue damage and restored cartilage homeostasis under polyphenol treatments [15,16].

Resveratrol is commonly recognized by the general public as an antioxidant in red wine, and it has been the most commonly investigated polyphenol in such studies. Resveratrol has shown anti-osteoarthritic effects due to its anti-apoptotic, anti-inflammatory and antioxidant properties [14]. Pycnogenol is a polyphenolic extract of the bark of French maritime pine (*Pinus maritima, Pinus pinaster*), and it has been shown to have antioxidative, anti-inflammatory and chondroprotective effects in vitro and in vivo. Moreover, in patients with osteoarthritis, clinical studies with Pycnogenol as an adjunct supplement have provided pain relief, the improvement of stiffness, and enhanced mobility [17]. Quercetin is one of the most abundant polyphenols in the ‘Mediterranean diet’, and it has also been shown to promote reduced inflammation and pain relief in animal models of osteoarthritis [18,19]. Belinal is also a commercially available polyphenolic extract that is isolated from the branches of silver fir (*Abies alba* L.). It has shown higher antioxidant activity in vitro than that of resveratrol, ascorbic acid and butylated hydroxytoluene, but similar activity to epigallocatechin gallate [20]. Abigenol is isolated from the bark of silver fir under the same conditions as Belinal (water, 100 °C). Its chemical composition shows a very similar polyphenolic profile to Belinal, and it has shown higher antioxidant activity than Pycnogenol [21]. The Belinal water-extractable fraction of silver fir wood branches shows in vitro and in vivo antioxidant activities due to lignans and some other compounds it contains. Furthermore, as the in vitro gastrointestinal digestion of the lignans in Belinal is not significant [20], it is reasonable to expect antioxidative effects from its oral application. Indeed, the effects of Belinal as a food supplement have been shown more recently for the prediabetic status, and it might be beneficial also in type 2 diabetes mellitus [22]. Belinal has also shown benefits in animal models of atherosclerosis [23,24], although its antioxidant activity has not yet been investigated in osteoarthritis.

Based on these data, we investigated whether the polyphenols in the Belinal extract can stimulate the in vitro chondrogenic potential of MSCs derived from patients with osteoarthritis. To this end, we initially screened for the effects of Belinal, the bark polyphenolic extract Pycnogenol, and the two most commonly used polyphenols, resveratrol and quercetin, on the viability and proliferation of MSCs. Secondly, we investigated the effects of selected concentrations of these two polyphenolic extracts and the two polyphenols on chondrogenesis under non-inflammatory and inflammatory conditions. These data show significant beneficial effects of Belinal on the chondrogenesis of MSCs derived from patients with osteoarthritis, under both non-inflammatory and inflammatory conditions. This study thus provides evidence that Belinal can be used to boost the chondrogenic potential of MSCs from patients with osteoarthritis, and it provides the basis for future in vitro and in vivo studies of the treatment of patients with this debilitating disorder.

## 2. Results

### 2.1. Determination of the Optimal Concentrations of the Polyphenolic Extracts and Polyphenols

**Figure 1:**
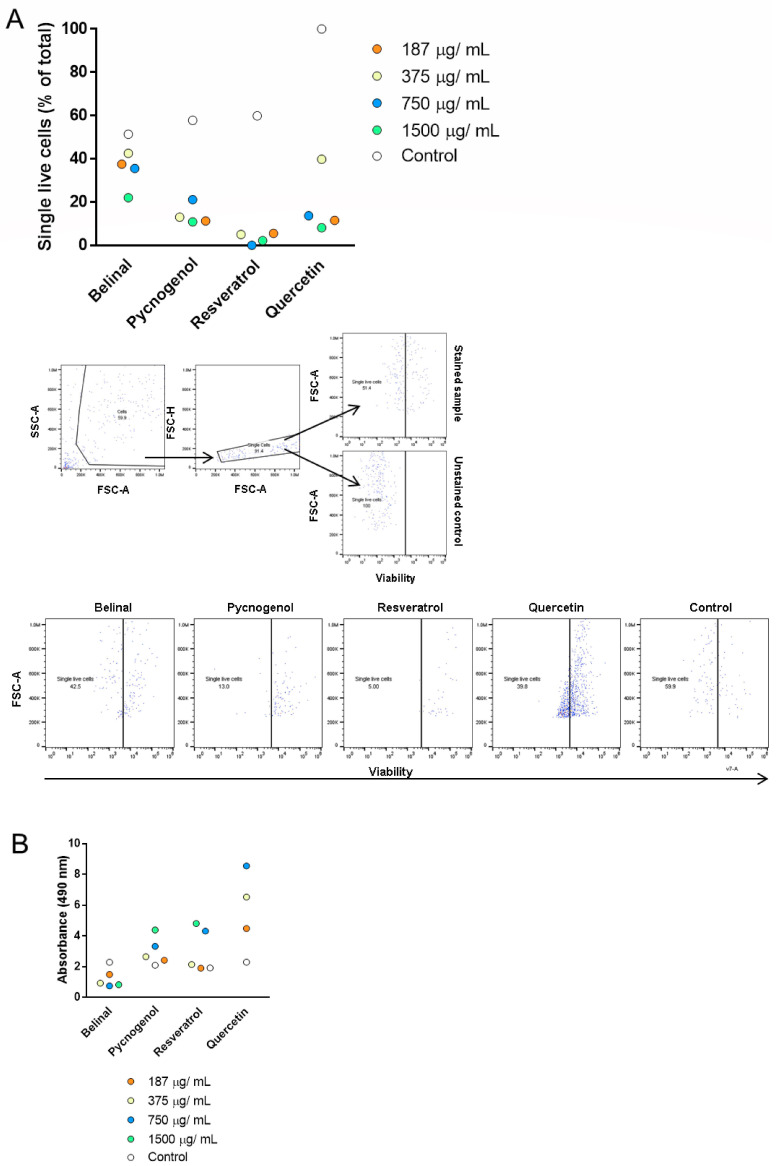
Viability screening to determine the optimal concentration of the polyphenolic extracts and polyphenols for further experiments. Viability of mesenchymal stem/stromal cells (MSCs) was determined for four serial dilutions (1500, 750, 375, 187 µg/mL) of the polyphenolic extracts and polyphenols (each dot showing the mean of triplicates) using flow cytometry (**A**) and the MTS assay (**B**) after 7 days of treatment. Controls were incubated in cell medium with no additions. (**A**) Top: proportions of single live cells. Middle: flow cytometry gating strategy. Bottom: representative dot plots for viability staining for each polyphenolic extract and polyphenol at 375 mg/mL. The highest concentration of the polyphenolic extracts and polyphenols (1500 µg/mL) showed significant reductions in the proportions of single live cells compared to controls (one-way ANOVA with Bonferroni correction). (**B**) Absorbance for the MTS assay measured at 490 nm. No significant differences are seen between the tested serial dilutions of the polyphenolic extracts and the polyphenols (one-way ANOVA with Bonferroni correction). Each concentration for each treatment was tested in triplicate. FSC-A, forward scatter area; SSC-A, side scatter area; FSC-H, forward scatter height.

Cell viability and proliferation were determined for four serial dilutions (1500, 750, 375, 187 µg/mL) of the polyphenolic extracts and polyphenols tested (performed in triplicate), using flow cytometry (Figure 1A) and the 3-(4,5-dimethylthiazol-2-yl)-5-(3-carboxymethoxyphenyl)-2-(4-sulfophenyl)-2H-tetrazolium (MTS) assay (Figure 1B). The controls included a cell medium with no treatments. The highest concentrations of the polyphenolic extracts and polyphenols used (1500 µg/mL) showed the significant reduction of single live mesenchymal stem/stromal cells (MSCs) cells compared to the controls (*p* = 0.0008; one-way ANOVA with Bonferroni correction). However, no significant differences were seen for cell proliferation between the tested serial dilutions of the polyphenolic extracts and the polyphenols (one-way ANOVA with Bonferroni correction). Based on these data, the concentration of 375 µg/mL was selected for further experiments.

### 2.2. Effects of the Polyphenolic Extracts and Polyphenols on Chondrogenesis under Non-Inflammatory Conditions

**Figure 2:**
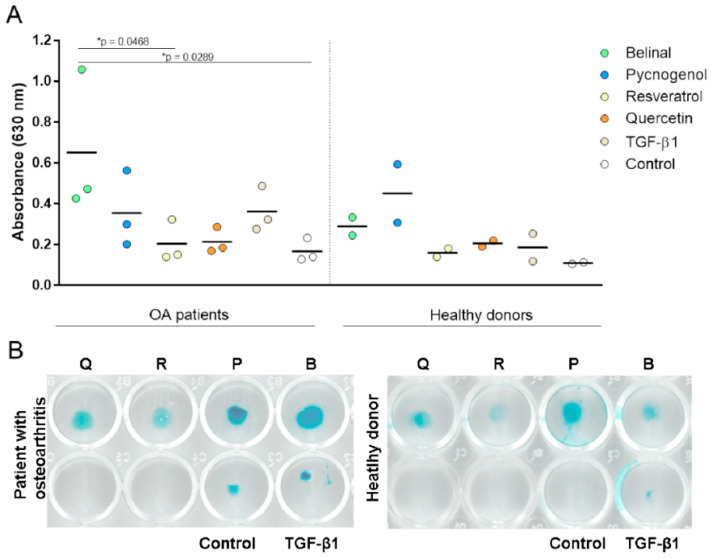
Chondrogenesis under non-inflammatory conditions. Chondrogenic differentiation was determined using the micromass assay. (**A**) Primary human (h)MSCs from patients with hip osteoarthritis (*n* = 3) and donors post mortem without musculoskeletal disorders (healthy donors, *n* = 2) were used to determine the effects of the polyphenolic extracts and polyphenols on chondrogenesis. Micromasses were treated with the polyphenolic extracts and polyphenols, with the addition of TGF-β1 for 7 days. Control micromasses received a medium without TGF-β1. Alcian blue staining was performed to visualize the glycosaminoglycans formed. The absorbance of the extracted Alcian blue was then measured at 630 nm, as the measure of chondrogenesis. Significant benefits were seen for Belinal over the control and resveratrol treatments, as indicated (one-way ANOVA with Bonferroni correction). (**B**) Representative scans of the Alcian blue-stained glycosaminoglycans in the micromasses. B, Belinal; P, Pycnogenol; R, resveratrol; Q, quercetin.

**Figure 3:**
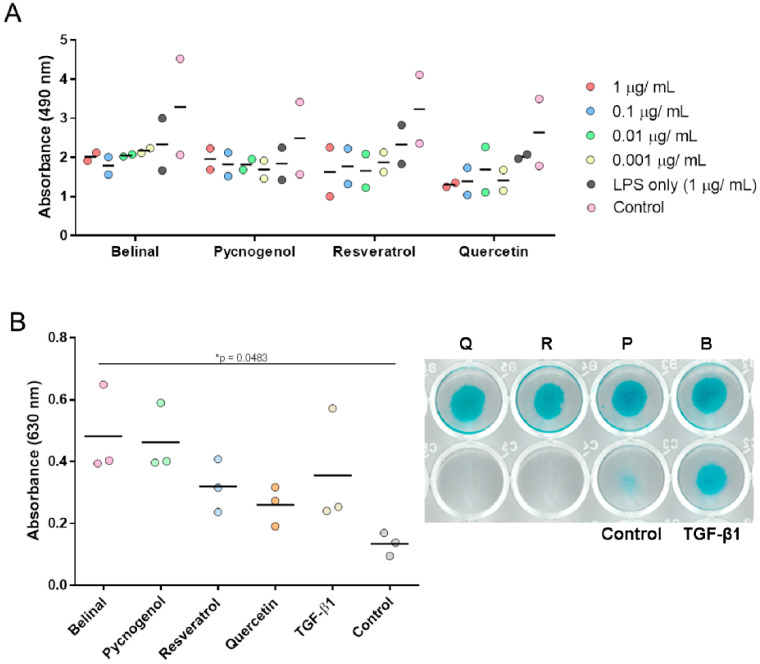
Chondrogenesis under inflammatory conditions. (**A**) Cell proliferation screening to determine the optimal lipopolysaccharide (LPS) concentration for further experiments, using the MTS assay. Serial dilutions of LPS (1, 0.1, 0.01, 0.001 µg/mL) were added to the primary hMSCs for 18 h, to simulate inflammatory conditions. After 18 h, the medium was changed and the polyphenolic extracts and polyphenols were added at 375 mg/mL. No significant differences in viability (as measured at 490 nm in the MTS assay) were seen between the serial dilutions of LPS. LPS at 0.01 µg/mL was used for further experiments based on previous studies [25,26]. Each concentration of LPS for each treatment was tested in duplicate. (**B**) Chondrogenic differentiation was determined using the hMSCs from patients with hip osteoarthritis (*n* = 3) in the micromass assay. Micromasses were pretreated with 0.01 µg/mL LPS for 18 h, and then treated with the polyphenolic extracts and polyphenols, with the addition of TGF-β1 for 7 days. After 7 days, Alican blue staining was performed as the measure of chondrogenesis. Belinal showed significantly greater absorbance of extracted Alcian blue in comparison with the controls that only received cell medium. B, Belinal; P, Pycnogenol; R, resveratrol; Q, quercetin.

Chondrogenic differentiation was determined using micromass assays and Alcian blue staining (Figure 2A). The primary human (h)MSCs from patients with hip osteoarthritis (*n* = 3) and donors post mortem without musculoskeletal disorders (i.e., healthy donors, *n* = 2) were used to determine the effects of the polyphenolic extracts and polyphenols on the chondrogenesis of MSCs. In the MSCs from patients with hip osteoarthritis, Belinal showed significantly higher absorbance of the extracted Alcian blue (and thus increased chondrogenesis) in comparison with the control and the polyphenol resveratrol (*p* = 0.0289, 0.0468, respectively; one-way ANOVA with Bonferroni correction). For the chondrogenesis of the MSCs derived from healthy donors, there were no significant differences seen between the different treatments (Figure 3A; one-way ANOVA with Bonferroni correction).

Representative scans for the Alcian blue stained glycosaminoglycans in the micromasses from the MSCs of patients with hip osteoarthritis and of healthy donors are shown in Figure 2B. For MSCs from patients with hip osteoarthritis, the Alcian blue staining (and thus chondrogenesis) was significantly greater in the micromasses treated with Belinal in comparison with the control and resveratrol-treated micromasses (Figure 3B, left). For the chondrogenesis of the MSCs of healthy donors, no significant differences were seen between the treatments with the polyphenolic extracts and polyphenols (Figure 3B, right).

### 2.3. Effects of the Polyphenolic Extracts and Polyphenols on Chondrogenesis under Inflammatory Conditions

Cell proliferation was screened to determine the optimal lipopolysaccharide (LPS) concentration for further experiments, using MTS assays (Figure 3A). No significant differences in cell proliferation were seen between the tested serial dilutions of LPS (Figure 3A). Based on these data and the results of previous studies using primary MSCs [25,26], 0.01 µg/mL LPS was selected for chondrogenesis under inflammatory conditions. In comparison with the control micromasses, the Alcian blue staining (and thus chondrogenesis) was significantly greater in the micromasses treated with Belinal (Figure 3B; *p* = 0.0483; one-way ANOVA with Bonferroni correction).

## 3. Discussion

Osteoarthritis is a widespread joint disease in the elderly population that has a substantial socio-economic burden. Stem cell exhaustion and the decreased potency of their regenerative potential have been defined as hallmarks of aging [5]. Decreases in the chondrogenic regenerative potential of bone-marrow-derived MSCs has also been proposed in osteoarthritis [12]. Strategies to boost the regenerative, and in particular the chondrogenic, potential of MSCs are being pursued in studies in regenerative medicine.

As inflammation constitutes the major part of the osteoarthritis pathology, compounds with well recognized anti-inflammatory properties are of particular interest for the treatment of osteoarthritis. Since phytochemicals in natural remedies can have anti-inflammatory, anti-oxidant and anabolic effects, much is to be expected from their chondro-inductive and chondroprotective effects for the treatment of osteoarthritis [14]. Polyphenols such as resveratrol, quercetin and those contained in the polyphenolic extract Pycnogenol, have all been shown to have beneficial effects in both in vitro and in vivo models of osteoarthritis [13,17,18,27,28]. Belinal is another commercially available polyphenolic extract, which is obtained from silver fir branches, and it has shown higher antioxidant activities (in ABTS assays) than resveratrol in vitro [20]. This water-extractable fraction of the silver fir branches shows in vitro and in vivo antioxidant activities due to lignans and to some other compounds it contains [20]. Abigenol is the commercial name for a chemically similar polyphenolic extract of silver fir bark, and it has shown higher antioxidant activity than Pycnogenol in vitro [21]. However, the antioxidant activities of these two silver fir extracts has not been established in osteoarthritis models to date.

In the present study, we investigated the effects of Belinal on the in vitro chondrogenesis of hMSCs derived from patients with osteoarthritis and from donors (post mortem) with no musculoskeletal disorders. The effects of Belinal were compared to those of the previously well recognized antioxidative and anti-inflammatory polyphenols resveratrol and quercetin, and the pine-bark polyphenolic extract Pycnogenol. We initially performed cell viability and proliferation screening to determine the optimal concentration of the polyphenolic extracts and polyphenols tested. Then, we performed in vitro chondrogenesis assays without and with pre-treatment with LPS, to simulate non-inflammatory and inflammatory conditions, respectively.

The data from the cell viability and proliferation screening indicated the use of 375 µg/mL as the optimal concentration of the polyphenolic extracts and polyphenols for chondrogenesis in this MSC model. Moreover, these data showed significant benefits of Belinal for in vitro chondrogenesis for the MSCs from patients with hip osteoarthritis under non-inflammatory conditions. Of interest, no such benefits were seen when the MSCs from healthy donors underwent the same treatments. This might be because bone-marrow-derived MSCs from patients with advanced osteoarthritis have reduced chondrogenic potential in comparison with healthy donors [12].

Previous studies have used resveratrol at concentrations as high as 100 µM (22.8 µg/mL) to show its anti-inflammatory effects on interleukin-1β-induced inflammation in primary human chondrocytes in vitro [14]. Pycnogenol at concentrations of up to 200 µg/mL has been shown to suppress pro-inflammatory responses in human chondrocytes and synovial fibroblasts in vitro [28]. Quercetin at 25 μM (7.6 µg/mL) has shown protective effects on *tert*-butyl hydroperoxide-stimulated rat chondrocytes, via the attenuation of oxidative stress and apoptosis [19]. In addition, 10 μM quercetin was shown to channel the differentiation of human bone-marrow-derived MSCs into adipocytes, rather than osteoblast bone cells [29].

The concentration of the polyphenolic extracts and polyphenols used in the present study was higher than those used in previous studies. However, the cell models used in previous studies were different from these trabecular bone-marrow-derived MSCs from patients with osteoarthritis. The most similar cell model was used in a study by Casado-Díaz et al. (2016), where they used bone-marrow-derived MSCs, although they obtained these from healthy volunteers [29]. It appears that the bone-derived MSCs in the present study required higher concentrations of the polyphenolic extracts and polyphenols for optimal effects, which might be due to the previously mentioned changes associated with stem cell exhaustion and reduced chondrogenic potential in osteoarthritis [11,12]. One of the possibilities to provide appropriate concentrations of these substances at sites of the damaged joints, and in particular in the cartilage, is the use of specific biomaterials such as silica aerogel. Indeed, it has been shown that resveratrol-loaded silica aerogel is biocompatible and stable, and that when it is coupled with the anti-inflammatory effects of resveratrol, it showed good potential for osteoarthritis treatment [30]. Based on our results, Belinal is a strong candidate to be tested with the same strategy.

Moreover, the screening of serial LPS dilutions in the present cell model did not show any significant differences in cell proliferation for the LPS treatments up to 1 µg/mL. Hence, the concentration of LPS for chondrogenesis under inflammatory conditions was selected based on previous studies using other cell models; e.g., 0.001 µg/mL LPS was used to pre-treat mouse macrophages, and 0.010 µg/mL LPS was used to pre-treat a murine microglial cell line [25,26]. The data for chondrogenesis under inflammatory conditions here (i.e., LPS pre-treatment of the micromasses) again showed the significant beneficial effects of Belinal, in comparison with the controls.

To sum up, using primary human MSCs from patients with osteoarthritis, we showed significant stimulation by Belinal of in vitro chondrogenesis in comparison with two polyphenols and a further polyphenolic extract with previously shown anti-inflammatory effects in in vitro models of osteoarthritis.

The main advantage of our study was the inclusion of the primary human MSCs from patients with advanced hip osteoarthritis as well as healthy donors with no musculoskeletal disorders. Although these represent in vitro models, they more closely resemble the endogenous cells that would be the real targets of these compounds in vivo. Therefore, our study provides useful in vitro data that underpin the need for further trials on the effects of Belinal in osteoarthritis.

There are at least two main disadvantages in this study. First, primary cell isolation was performed from different joints in the patients with osteoarthritis and healthy donors. This was due to the lack of ethical consent to sample hip tissue in post mortem donors, as knee is more easily accessed during autopsies. Secondly, we did not test the primary human MSCs from healthy donors under inflammatory conditions. Larger numbers of repeat experiments should be carried out on a larger scale with healthy donors and patients to be able to provide stronger evidence of the positive effects of the Belinal on chondrocyte differentiation. Moreover, the variance between the different donors, as already observed, is to be expected, as the cell populations from different donors might very well differ. The higher number of donors and the analysis of the cellular compositions of MSC populations would strengthen these studies.

Further studies are also needed to highlight the influence of Belinal in inflammatory disorders, e.g., rheumatoid arthritis.

## 4. Materials and Methods

**Figure 4:**
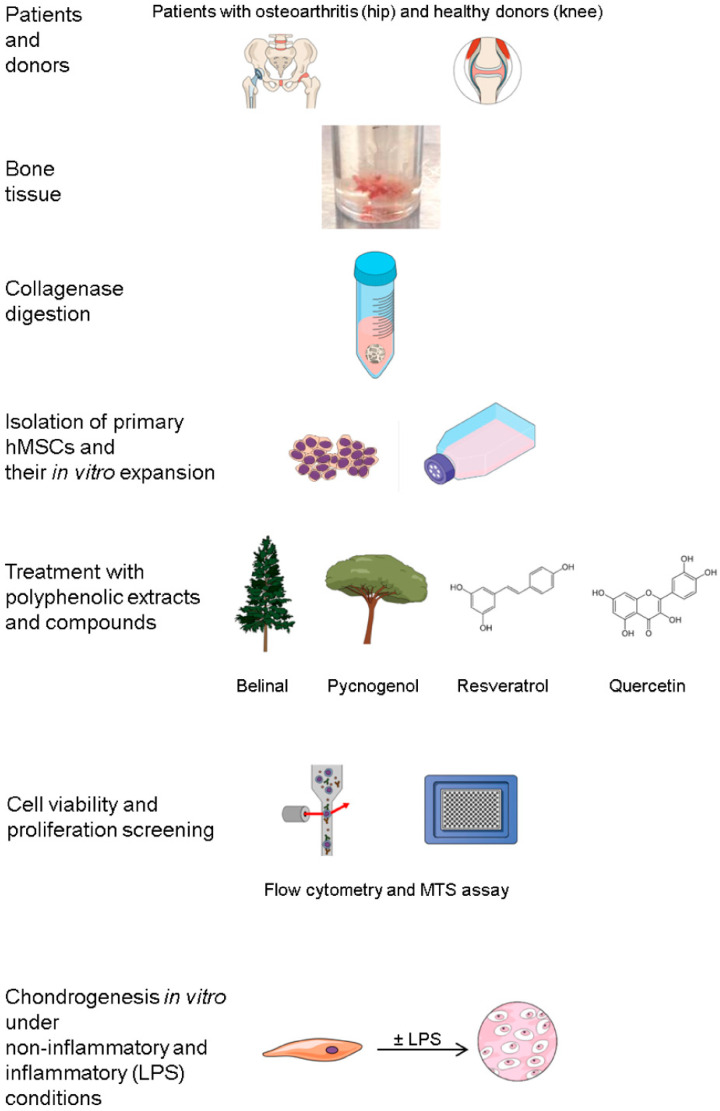
Study design and primary human (h)MSC samples. Primary hMSCs were isolated from the trabecular bone of the femoral head from patients with osteoarthritis undergoing routine hip replacement surgery, and from the medial tibia from donors post mortem with no musculoskeletal disorders who had routine autopsies. The MSC-like phenotype for these samples was confirmed previously [31]. The in vitro expanded cells from passages 3 to 5 were used in the experiments, initially in assays of cell viability and in proliferation screening for the determination of the optimal concentration of the polyphenolic extracts (Belinal, Pycnogenol) and polyphenols (resveratrol, quercetin) (flow cytometry and MTS assay). These MSCs were then used to investigate chondrogenic differentiation, to determine whether the polyphenolic extracts and polyphenols can support the in vitro chondrogenic differentiation of primary hMSCs from osteoarthritis patients under non-inflammatory and inflammatory (+lipopolysaharide (LPS)) conditions.

The study design and the analyses are summarised in Figure 4.

### 4.1. Ethics Statement and Patient Selection

The primary hMSCs used in this study were derived from three patients with hip osteoarthritis who were undergoing routine total hip arthroplasty at the Valdoltra Orthopaedic Hospital, Ankaran, Slovenia. Hip osteoarthritis was diagnosed by clinical examinations and X-rays. The exclusion criteria included a history of inflammatory arthritis, metastatic cancer and disorders that can affect the bone. Two donors with no macroscopic evidence or history of musculoskeletal disorder (i.e., healthy donors) who had routine autopsies carried out at the Institute of Forensic Medicine, Ljubljana, Slovenia, were also included in the study. Trabecular bone was sampled from the femoral head in the patients with osteoarthritis, and from the medial tibia in the healthy (post mortem) donors. Approval for this study was obtained from the National Medical Ethics Committee of the Republic of Slovenia (reference numbers: 0120-523/2016-2, KME 45/10/16, 0120-523/2016/11). Written informed consent to participate in this study was obtained from all patients.

### 4.2. Isolation of Primary hMSCs and Culture Conditions

Primary hMSCs from trabecular bone were isolated as described previously [31]. Briefly, the bone biopsies from the femoral head were digested in 1 mg/mL collagenase (Roche, Basel, Switzerland) at 37 °C for 3 h. The digested tissue suspension was filtered through a 70 µm nylon strainer (Corning, New York, NY, USA). The cells were seeded in 1.0 mL MSC expansion medium (Kit XF, human; Miltenyi Biotec, Bergisch Gladbach, Germany) supplemented with 2% penicillin and streptomycin (100× stock; 8.5 g/L sodium chloride, 0.025 g/L amphotericin B, 6.028 g/L penicillin G sodium salt, 10 g/L streptomycin sulphate; Biowest, Nuaillé, France), and 2 mM glutamine (all Biowest, Nuaillé, France), and maintained at 37 °C in a 5%/5% humidified CO_2_/O_2_ atmosphere. The cells were culture expanded, and cells at passages 3 to 5 were used for all of the experiments. The MSC-like phenotype of these primary hMSCs was confirmed prior to the experiments, according to the International Society for Cellular Therapy [32], as previously reported [31].

### 4.3. Polyphenolic Extract and Polyphenol Preparation

The standardised plant polyphenolic extracts Belinal and Pycnogenol were obtained from Abies Labs and Plantex, respectively, and the purified polyphenols resveratrol (catalogue number R5010) and quercetin (catalogue number Q4951) were from Sigma-Aldrich (St. Louis, MO, USA). Phytochemical investigations of Belinal [21] and Pycnogenol [17] used in this study have been performed previously. Briefly, the Belinal water extract of silver fir (*Abies alba*) wood contains lignans, which constitute approximately 10% of the extract and include isolariciresinol, hydroxymatairesinol, secoisolariciresinol, lariciresinol, pinoresinol, and matairesinol [21]. All four of the tested substances were prepared as stock solutions at 10.0 mg/mL, with Belinal and Pycnogenol dissolved in ultrapure sterile water, and resveratrol and quercetin dissolved in 96% ethanol (Merck Milipore, Burlington, MA, USA). The working solution used for the cells contained <0.5% ethanol.

### 4.4. Flow Cytometry and Cell Proliferation Assays for Viability Screening

Primary hMSCs were seeded at 2000 cells/well in two 96-well plates, with four replicates per plate. Viability screening was performed for four serial dilutions of the two polyphenolic extracts and the two polyphenols, as 1500, 750, 375 and 187 µg/mL. The cells were treated for 7 days, with fresh dilutions of the polyphenolic extracts and polyphenols prepared every second day. The negative controls included cells treated with the culture medium, and also cells treated with culture medium with the addition of the treatment solvents (i.e., ultrapure water, 96% ethanol), in two replicates. 

For flow cytometry, the cells were detached (0.25% trypsin–EDTA; Biowest, Nuaillé, France) and the cell suspensions were stained using the fixable viability dye eFluor 780 (ThermoFisher Scientific, Waltham, MA, USA). Data were acquired using a flow cytometer (Attune NxT; ThermoFisher Scientific, Waltham, MA, USA), with a recording of at least 10,000 single live cells. The unstained control included the same cells without the addition of viability dye, and was used to set up the gate. The data were analysed using the FlowJo v10 software (Ashland, OR, USA).

To monitor cell proliferation, a commercial cell proliferation assay was used (CellTiter 96 Aqueous One Solution; Promega, Madison, WI, USA), according to the manufacturer instructions. The reagent was added to the culture wells and incubated at 37 °C for 4 h. Absorbance was measured at 490 nm using a microplate reader (Synerg H4 Hybrid Multi-Mode; BioTek, Winooski, VT, USA).

### 4.5. Chondrogenic Differentiation

Chondrogenic differentiation was carried out using micromass cultures for all three of the donors under both physiological and inflammatory conditions. For the induction of inflammatory conditions, LPS was used as a general activator of proinflammatory cytokines, instead of a more specific cytokine activator like IL-1 or TNF-α. The optimal concentration of LPS from *Escherichia coli* (O111:B4; Sigma-Aldrich, St. Louis, MO, USA) was determined using MTS cell viability assays. Micromasses were formed using 150,000 cells in 10 µL serum-free medium that consisted of high-glucose Dulbecco’s modified Eagle’s medium (Biowest, Nuaillé, France), 100 nM dexamethasone (Sigma-Aldrich, St. Louis, MO, USA), 1% insulin–transferrin–selenium (Sigma, St. Louis, MO, USA), 50 mg/mL ascorbic acid-2-phosphate (Sigma, St. Louis, MO, USA), and 2% penicillin and streptomycin (100× stock; 8.5 g/L sodium chloride, 0.025 g/L amphotericin B, 6.028 g/L penicillin G sodium salt, 10 g/L streptomycin sulphate; Biowest, Nuaillé, France). The micromasses were allowed to attach to the well surface of 24-well plates pre-covered with 1.0 g/L gelatine (Sigma, St. Louis, MO, USA) for 45 min. Then, 0.5 mL of serum-free medium was added. The next day, chondrogenic medium was added to each well, which consisted of the same serum-free medium with the addition of 10 ng/mL transforming growth factor (TGF)-β1 (ThermoFisher Scientific, Waltham, MA, USA), and with the addition of each polyphenolic extract or polyphenol to be tested. The controls comprised micromasses without TGF-β1 and control micromasses with TGF-β1 but without any polyphenolic extracts or polyphenols. The assays were carried out for 7 days, with regular changes of the medium with freshly prepared medium, as before.

Chondrogenesis was assessed using Alcian blue staining (Sigma-Aldrich, St. Louis, MO, USA). The micromasses were fixed using ice-cold methanol (Merck Milipore, Burlington, MA, USA) for 30 min at −20 °C. The Alcian blue was filtered and 500 µL was added per micromass, for 12 h. The micromasses were washed three times with distilled water and the 24-well plates were scanned to visualise the stained micromasses. The Alcian blue was then extracted with 100 µL 6 M guanidine hydrochloride (Sigma-Aldrich, St. Louis, MO, USA) in distilled water, over 6 h at room temperature, with gentle rocking. The extracted solutions were transferred (100 µL) to 96-well plates. Guanidine hydrochloride (Sigma-Aldrich, St. Louis, MO, USA) was included as the blank. The absorbance of the extracted Alcian blue was measured at 630 nm using a microplate reader (Tecan Safire2; Biotek, Winooski, VT, USA).

### 4.6. LPS-Induced Inflammation

Lipopolysaccharide from *E. coli* O111:B4 (Sigma, St. Louis, St. Louis, MO, USA) was dissolved in phosphate-buffered saline at the stock concentration of 1 mg/mL. The cell proliferation screening to determine the optimal LPS concentration for further experiments was performed using MTS assays. Serial dilutions of LPS (1, 0.1, 0.01, 0.001 µg/mL) were added to primary hMSCs from the patients with hip osteoarthritis, which were left for 18 h to simulate inflammatory conditions. After 18 h, the cell medium was changed and the tested polyphenolic extracts and polyphenols were added at 375 mg/mL. The MTS assays were performed as described above. Based on the results of these MTS assays, the optimal concentration of LPS was selected as 0.01 µg/mL for the condition of chondrogenesis under inflammation.

Therefore, micromasses were formed from MSCs derived from the patients with hip osteoarthritis and were pre-treated with 0.01 µg/mL LPS for 18 h to simulate inflammation. Then, the micromasses were treated with the polyphenolic extracts and polyphenols to be tested, and with the addition of TGF-β1, for 7 days. Alcian blue was used to determine chondrogenesis as described above.

### 4.7. Statistical Analysis

To compare the data between the polyphenolic extracts, Belinal and Pycnogenol and the polyphenols resveratrol and quercetin, either one-way ANOVA or two-way ANOVA with Bonferroni post hoc for multiple testing was used. The statistical analyses were performed using GraphPadPrism, version 6 for Windows (GraphPad Software, La Jolla, CA, USA, www.graphpad.com). *p* values < 0.05 were considered as statistically significant. The Figures were created using Mind the Graph (https://mindthegraph.com).

## 5. Conclusions

In conclusion, these data show significant beneficial effects of Belinal, a water-extractable fraction of silver fir branches, on in vitro chondrogenesis of MSCs derived from patients with osteoarthritis, under both non-inflammatory and inflammatory conditions. This study thus provides evidence that Belinal can be used to boost the in vitro chondrogenic potential of MSCs from patients with osteoarthritis and suggests further in vitro and in vivo studies using this polyphenolic extract.

## References

[1] March L., Cross M., Arden N., Hawker G. Osteoarthritis: A Serious Disease. https://research-information.bris.ac.uk/en/publications/osteoarthritis-a-serious-disease-submitted-to-the-us-food-and-dru.

[2] Chen D., Shen J., Zhao W., Wang T., Han L., Hamilton J.L., Im H.-J. (2017). Osteoarthritis: Toward a comprehensive understanding of pathological mechanism. Bone Res..

[3] Rai V., Dilisio M.F., Dietz N.E., Agrawal D.K. (2017). Recent strategies in cartilage repair: A systemic review of the scaffold development and tissue engineering. J. Biomed. Mater. Res. Part A.

[4] Kapoor M., Martel-Pelletier J., Lajeunesse D., Pelletier J.P., Fahmi H. (2011). Role of proinflammatory cytokines in the pathophysiology of osteoarthritis. Nat. Rev. Rheumatol..

[5] De Bari C., Roelofs A.J. (2018). Stem cell-based therapeutic strategies for cartilage defects and osteoarthritis. Curr. Opin. Pharmacol..

[6] Čamernik K., Barlič A., Drobnič M., Marc J., Jeras M., Zupan J. (2018). Mesenchymal stem cells in the musculoskeletal system: From animal models to human tissue regeneration?. Stem Cell Rev. Rep..

[7] Roelofs A.J., Zupan J., Riemen A.H.K., Kania K., Ansboro S., White N., Clark S.M., De Bari C. (2017). Joint morphogenetic cells in the adult synovium. Nat. Commun..

[8] Harrell C.R., Markovic B.S., Fellabaum C., Arsenijevic A., Volarevic V. (2019). Mesenchymal stem cell-based therapy of osteoarthritis: Current knowledge and future perspectives. Biomed. Pharmacother..

[9] Samuel S., Ahmad R.E., Ramasamy T.S., Manan F., Kamarul T. (2018). Platelet rich concentrate enhances mesenchymal stem cells capacity to repair focal cartilage injury in rabbits. Injury.

[10] Owston H., Giannoudis P.V., Jones E. (2016). Do skeletal muscle MSCs in humans contribute to bone repair? A systematic review. Injury.

[11] Partridge L., Deelen J., Slagboom P.E. (2018). Facing up to the global challenges of ageing. Nature.

[12] Murphy J.M., Dixon K., Beck S., Fabian D., Feldman A., Barry F. (2002). Reduced chondrogenic and adipogenic activity of mesenchymal stem cells from patients with advanced osteoarthritis. Arthritis Rheum..

[13] Oliviero F., Scanu A., Zamudio-Cuevas Y., Punzi L., Spinella P. (2018). Anti-inflammatory effects of polyphenols in arthritis. J. Sci. Food Agric..

[14] Buhrmann C., Honarvar A., Setayeshmehr M., Karbasi S., Shakibaei M., Valiani A. (2020). Herbal remedies as potential in cartilage tissue engineering: An overview of new therapeutic approaches and strategies. Molecules.

[15] Leong D.J., Choudhury M., Hanstein R., Hirsh D.M., Kim S.J., Majeska R.J., Schaffler M.B., Hardin J.A., Spray D.C., Goldring M.B. (2014). Green tea polyphenol treatment is chondroprotective, anti-inflammatory and palliative in a mouse posttraumatic osteoarthritis model. Arthritis Res. Ther..

[16] Zhang Z., Leong D.J., Xu L., He Z., Wang A., Navati M., Kim S.J., Hirsh D.M., Hardin J.A., Cobelli N.J. (2016). Curcumin slows osteoarthritis progression and relieves osteoarthritis-associated pain symptoms in a post-traumatic osteoarthritis mouse model. Arthritis Res. Ther..

[17] Rohdewald P.J. (2018). Review on Sustained Relief of Osteoarthritis Symptoms with a Proprietary Extract from Pine Bark, Pycnogenol. J. Med. Food.

[18] Britti D., Crupi R., Impellizzeri D., Gugliandolo E., Fusco R., Schievano C., Morittu V.M., Evangelista M., Di Paola R., Cuzzocrea S. (2017). A novel composite formulation of palmitoylethanolamide and quercetin decreases inflammation and relieves pain in inflammatory and osteoarthritic pain models. BMC Vet. Res..

[19] Feng K., Chen Z., Pengcheng L., Zhang S., Wang X. (2019). Quercetin attenuates oxidative stress-induced apoptosis via SIRT1/AMPK-mediated inhibition of ER stress in rat chondrocytes and prevents the progression of osteoarthritis in a rat model. J. Cell. Physiol..

[20] Benković E.T., Žigon D., Mihailović V., Petelinc T., Jamnik P., Kreft S. (2017). Identification, *in-vitro* and *in-vivo* antioxidant activity, and gastrointestinal stability of lignans from silver fir (*Abies alba*) Wood Extract Belinal. J. Wood Chem. Technol..

[21] Benković E.T., Grohar T., Žigon D., Švajger U., Janeš D., Kreft S., Štrukelj B. (2014). Chemical composition of the silver fir (*Abies alba*) bark extract Abigenol^®^ and its antioxidant activity. Ind. Crops Prod..

[22] Debeljak J., Ferk P., Čokolič M., Zavratnik A., Benković E.T., Kreft S., Štrukelj B. (2016). Randomised, double blind, cross-over, placebo and active controlled human pharmacodynamic study on the influence of silver fir wood extract (Belinal) on post-prandial glycemic response. Pharmazie.

[23] Drevenšek G., Lunder M., Benković E.T., Štrukelj B., Kreft S. (2016). Cardioprotective effects of silver fir (*Abies alba*) extract in ischemic-reperfused isolated rat hearts. Food Nutr. Res..

[24] Drevenšek G., Lunder M., Benković E.T., Mikelj A., Štrukelj B., Kreft S. (2015). Silver fir (*Abies alba*) trunk extract protects guinea pig arteries from impaired functional responses and morphology due to an atherogenic diet. Phytomedicine.

[25] Hu Y., Qin C., Zheng G., Lai D., Tao H., Zhang Y., Qiu G., Ge M., Huang L., Chen L. (2016). Mesenchymal stem cell-educated macrophages ameliorate LPS-induced systemic response. Mediat. Inflamm..

[26] Liu Y., Zhang R., Yan K., Chen F., Huang W., Lv B., Sun C., Xu L., Li F., Jiang X. (2014). Mesenchymal stem cells inhibit lipopolysaccharide-induced inflammatory responses of BV2 microglial cells through TSG-6. J. Neuroinflamm..

[27] Lei M., Liu S., Liu Y. (2018). Resveratrol protects bone marrow mesenchymal stem cell derived chondrocytes cultured on chitosan-gelatin scaffolds from the inhibitory effect of interleukin-1β. Acta Pharmacol. Sin..

[28] Peng Y.J., Lee C.H., Wang C.C., Salter D.M., Lee H.S. (2012). Pycnogenol attenuates the inflammatory and nitrosative stress on joint inflammation induced by urate crystals. Free Radic. Biol. Med..

[29] Casado-Díaz A., Anter J., Dorado G., Quesada-Gómez J.M. (2016). Effects of quercetin, a natural phenolic compound, in the differentiation of human mesenchymal stem cells (MSCs) into adipocytes and osteoblasts. J. Nutr. Biochem..

[30] Qin L., He Y., Zhao X., Zhang T., Qin Y., Du A. (2020). Preparation, characterization, and *in-vitro* sustained release profile of resveratrol-loaded silica aerogel. Molecules.

[31] Čamernik K., Mihelič A., Mihalič R., Marolt Presen D., Janež A., Trebše R., Marc J., Zupan J. (2019). Skeletal-muscle-derived mesenchymal stem/stromal cells from patients with osteoarthritis show superior biological properties compared to bone-derived cells. Stem Cell Res..

[32] Dominici M., Le Blanc K., Mueller L., Slaper-Cortenbach I., Marini F., Krause D., Deans R., Keating A., Prockop D., Horwitz E. (2006). Minimal criteria for defining multipotent mesenchymal stromal cells. The International Society for Cellular Therapy position statement. Cytotherapy.

